# Escape from X chromosome inactivation and female bias of autoimmune diseases

**DOI:** 10.1186/s10020-020-00256-1

**Published:** 2020-12-09

**Authors:** Mohammad Javad Mousavi, Mahdi Mahmoudi, Somayeh Ghotloo

**Affiliations:** 1 Department of Hematology, Faculty of Allied Medicine, Bushehr University of Medical Sciences, Bushehr, Iran; 2 Department of Immunology, School of Medicine, Iran University of Medical Sciences, Tehran, Iran; 3 Rheumatology Research Center, Tehran University of Medical Sciences, Tehran, Iran; 4 Department of Medical Laboratory Sciences, School of Allied Medical Sciences, Kashan University of Medical Sciences, Kashan, Iran

**Keywords:** Autoimmune diseases, Escape genes, Female bias, X-chromosome inactivation

## Abstract

Generally, autoimmune diseases are more prevalent in females than males. Various predisposing factors, including female sex hormones, X chromosome genes, and the microbiome have been implicated in the female bias of autoimmune diseases. During embryogenesis, one of the X chromosomes in the females is transcriptionally inactivated, in a process called X chromosome inactivation (XCI). This equalizes the impact of two X chromosomes in the females. However, some genes escape from XCI, providing a basis for the dual expression dosage of the given gene in the females. In the present review, the contribution of the escape genes to the female bias of autoimmune diseases will be discussed.

## Background

Generally, autoimmune diseases are more prevalent in females than males. Various predisposing factors, including female sex hormones, X chromosome genes, and the microbiome have been implicated in the female bias of autoimmune diseases. During embryogenesis, one of the X chromosomes in the females is transcriptionally inactivated, in a process called X chromosome inactivation (XCI). This equalizes the impact of two X chromosomes in the females. However, some genes escape from XCI, providing a basis for the dual expression dosage of the given gene in the females. In the present review, the contribution of some escape genes to the female bias of autoimmune diseases was discussed.

The current literature provides evidence in the contribution of a number of the escape genes, including CD40L, CD99, LAMP-2, IRAK-1, TLR7, USP27X, DDX3X, CXORF21 and XIAP, in the autoimmunity. Current literature also confirms contribution of some escapes genes including CD40L, IRAK-1, TLR7, CXORF21 and XIAP to the female bias of autoimmune diseases especially SLE disease. However, more studies are required to evaluate contribution of these genes in the female bias of SLE using animal model of the disease and the efficacy of therapeutic interventions against these molecules. Next experiments are also necessary to address attribution of these genes to the female bias of other autoimmune diseases rather than SLE. Finally, investigating role of the other escape genes such as USP27X and DDX3X in the sex-bias of autoimmune diseases such as SLE, RA, MS may help to discover further mechanisms involved in the female bias of autoimmunity and consequently in developing more effective treatments and sensitive diagnostics of patients with autoimmune diseases.

## X chromosome inactivation (XCI)

The hypothesis of XCI was introduced by Mary Lyon in 1961. In the XCI process, one of the X chromosomes in women is inactivated in which DNA is tightly condensed into the heterochromatin structure and becomes transcriptionally inactive. As a result, XCI equalizes the impact of the presence of two X chromosomes in females (Disteche and Berletch 2015). XCI is a random process in which one of the X chromosomes during embryogenesis is randomly selected and inactivated (Patrat et al. 2013). It is an irreversible process and, the selected allele will be remained inactive throughout the life span (Patrat et al. 2013).

**Fig. 1:**
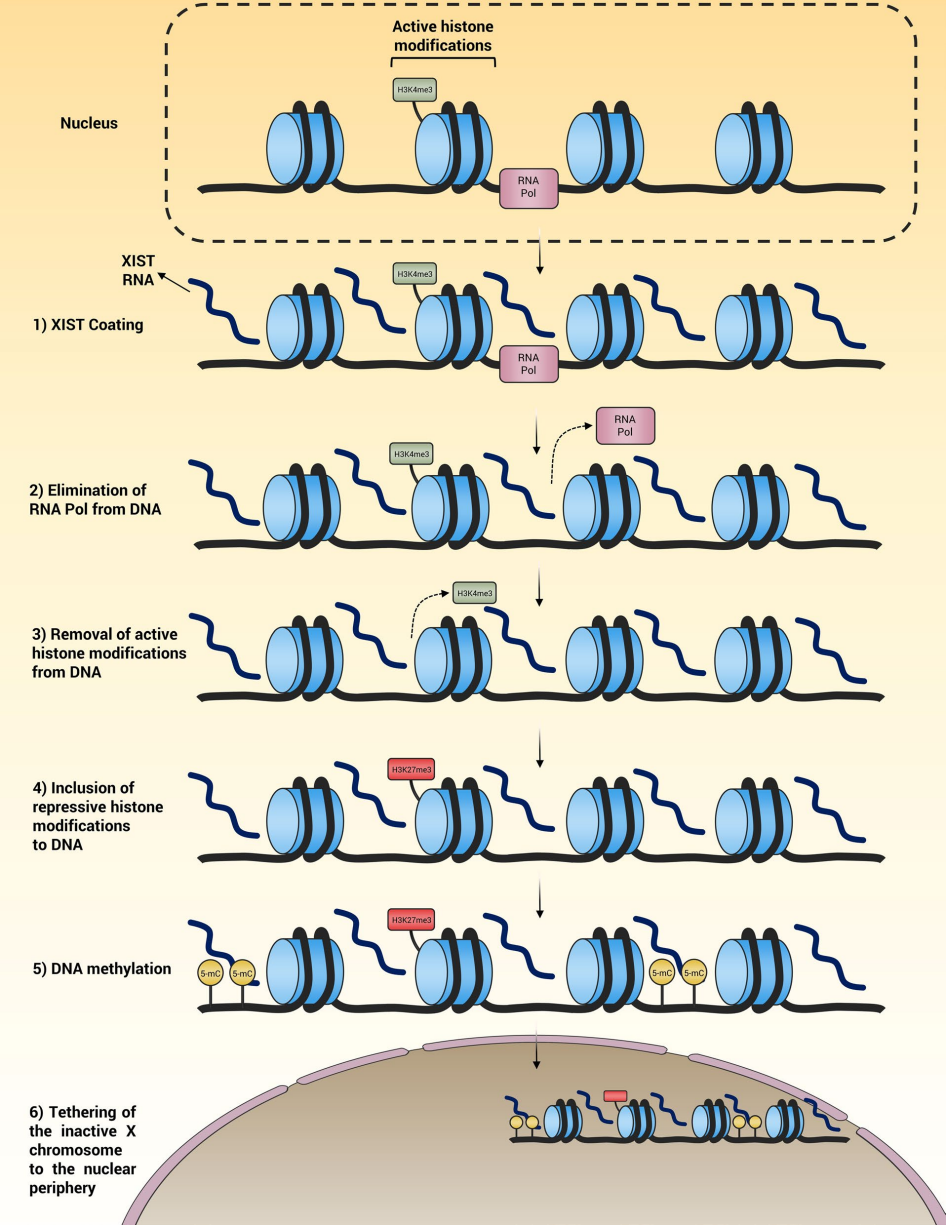
Contribution of XIST in XCI. XIST coats the chromosome and mediates various aspects of XIC, including elimination of RNA polymerases from DNA, removal of the active histone modifications from the chromosome, inclusion of the repressive histone modifications into the chromosome, methylation of DNA and tethering of the inactive X chromosome to the nuclear periphery. DNA: Deoxyribonucleic acid; XCI: X chromosome inactivation; XIST: X-inactive specific transcript; RNA: Ribonucleic acid

XCI is a regulated multi-step process initiated and continued by the following steps (1) Enumeration of X chromosomes (2) Selection of one chromosome for the XCI process (3) Initiation of XCI process (4) maintenance of XCI in the subjected genes (Lu et al. 2017). XCI process is started and controlled from X-inactivation center (XIC) in which a long non-coding ribonucleic acid (lncRNA) designated X-inactive specific transcript (XIST), plays a central role in the process. XIST mediates all aspects of XCI, including coating of the chromosome, elimination of RNA polymerases from deoxyribonucleic acid (DNA), removal of the active histone modifications allowing for transcription from DNA, inclusion of the repressive histone modifications into DNA resulting in transcriptional repression, methylation of DNA and tethering of the inactive X chromosome to the nuclear periphery (Fig. 1) (Lu et al. 2017).

XCI is initiated by binding of XIST to XIC on the X-chromosome and subsequent spreading along the chromosome. Minimal diffusion to the nucleoplasm occurs, ensuring the silencing does not spill over to the other chromosomes (Lu et al. 2017). In addition to XIST, various proteins which bind to the distinct sequence regions of XIST, cooperate with it in XCI process. Heterogeneous nuclear ribonucleoprotein U family of the proteins are implicated in XIST and coat the intended X chromosome for XCI (Lu et al. 2017). The polycomb repressive complexes (PRCs) proteins, including PRC1 and PRC2, play crucial roles in the transcriptional silencing through promotion of various histone modifications, including H2AK119 mono-ubiquitination (H2AK119ub) and H3K27 trimethylation (H3K27me3). These histone modifications exert transcriptional repression of the chromosome (Tamburri 2020; Pan et al. 2018). Lamin B receptor protein may mediate tethering of the inactivated X chromosome to the nuclear periphery appeared as the Barr body, the inactive X chromosome in the cell with more than one X chromosome (Lu et al. 2017).

## Escape from XCI

Evaluation of inactive X chromosomes in humans shows approximately 15% of the genes regularly escape from XCI, while around 15% of the other genes variably escape, as XCI escape is permanent in some genes (15%) and spatial in the others (15%) (Posynick and Brown 2019). The escaped genes are different among various tissues and individuals. This renders multiple tissues and individuals to be different in the extent of susceptibility to the diseases if the escaped gene is implicated in the disease pathogenesis (Peeters et al. 2014). Besides, a mosaics pattern of XCI either in paternal or maternal X chromosome is observed in the cells of female body (Posynick and Brown 2019).

Conventionally, X chromosome genes with a homologous gene on the Y chromosome have been considered to escape from XCI, allowing for the dosage compensation in the females (Posynick and Brown 2019). However, almost many escape genes are devoid of a functional homologue on the Y chromosome. Some of these genes render the female gender with a specific benefit. For example, these genes are implicated in the success of the pregnancy (Posynick and Brown 2019). The neighborhood elements of a gene also contribute to its inactivation or escape pattern. An enrichment of Alu elements and the other simple repeats was identified at approximate of the escape genes, while L1 and L2 types of long interspersed nuclear elements, were associated with the genes subjected to XCI (Posynick and Brown 2019; Peeters et al. 2014). Moreover, the presence of the boundary elements between escape and subject genes may prevent spreading of the silencing into the neighboring regions. The regulatory role of CCCTC-binding factor, a transcription factor involved in the transcriptional regulation, insulator activity and regulation of three-dimensional structure of chromatin (Kim et al. 2015), in the boundary regions has been identified (Posynick and Brown 2019; Peeters et al. 2014).

**Fig. 2:**
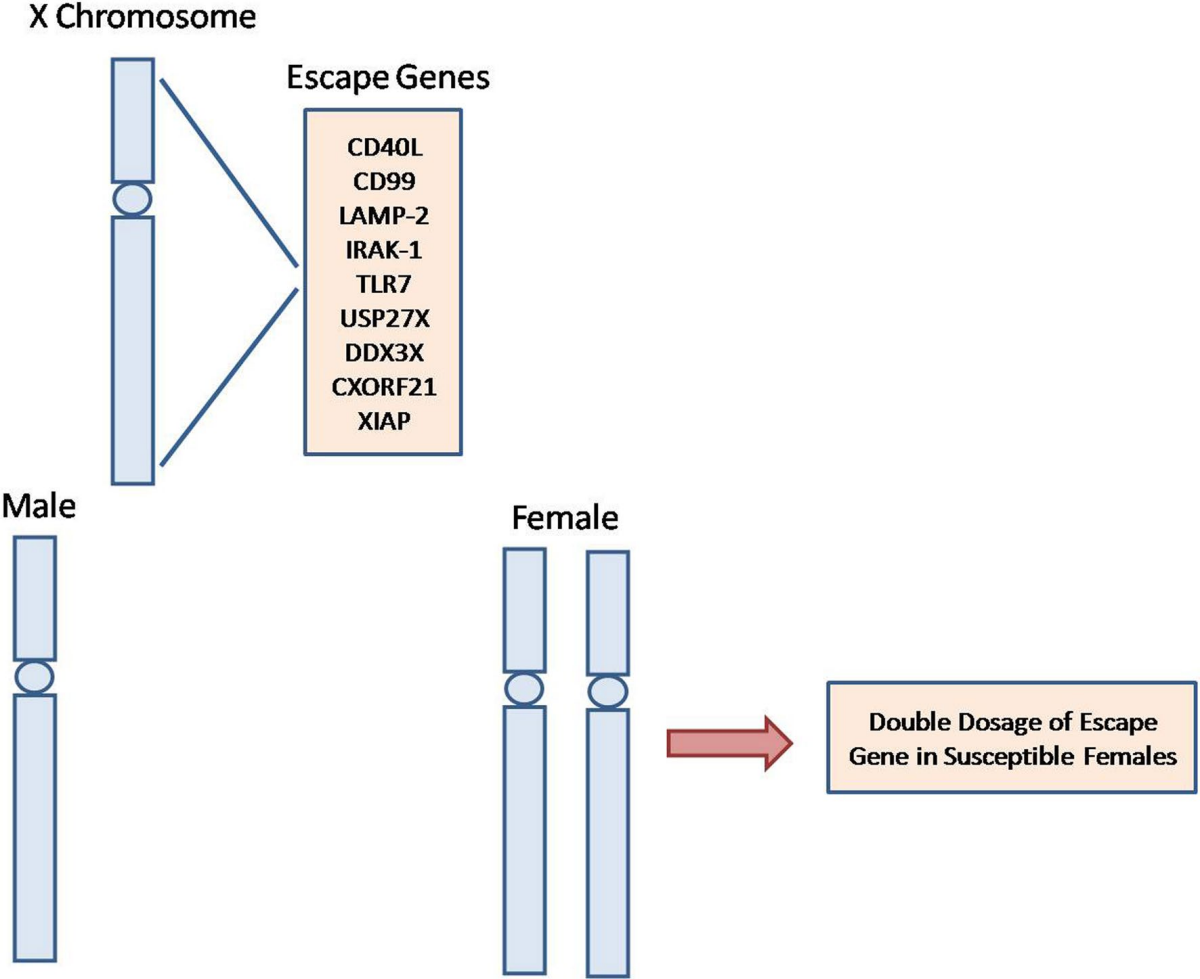
A schematic diagram of contribution of escape genes to the female bias of autoimmune diseases. Prone females can express the escape genes in double doses compared to males due to its expression from two X chromosome. CD40L: Cluster of differentiation 40 ligand; CD99: Cluster of differentiation 99; CXORF21: Chromosome X open reading frame 21; IRAK-1: Interleukin-1 receptor-associated kinase-1; XIAP: X-linked inhibitor of apoptosis protein; TLR7: Toll-like receptor 7; USP27X: Ubiquitin carboxyl-terminal hydrolase 27

A recent report by Katsir et al. identified a number of 24 genes, corresponding to 10.3% of all expressed genes in the X chromosome, which escape from XCI (Wainer Katsir and Linial 2019). The others cited another set of the escape genes (Odhams 2019; Carrel and Willard 1999; Wang 2016). A summary of these genes mostly involved in innate and adaptive immune response is given in Table 1. XCI escape may provide a basis for more susceptibility of women than men to the autoimmune diseases (Fairweather and Rose 2004), as the prone women can produce a given pathogenic protein in double doses than men due to its expression from two X chromosome (Fig. 2). In the following sections, the contribution of the genes with the possible implications in autoimmune diseases will be reviewed.

**Table 1:** A list of the genes escaped from X chromosome inactivation

-	Gene symbol	Gene nomenclature	Aliases for the gene	Reference numbers
1	CD99	Cluster of differentiation 99	HBA71, MIC2, MIC2X, MIC2Y, MSK5X, Xg blood group	Wainer Katsir and Linial (2019)
2	LAMP2	Lysosomal associated membrane protein 2	CD107b, LAMPB, LGP110, LGP-96, DND	Wainer Katsir and Linial (2019)
3	USP27X	Ubiquitin specific peptidase 27 X-linked	Deubiquitinating enzyme 27, USP22L, USP27, Ubiquitin Carboxyl-Terminal Hydrolase 22-Like, Ubiquitin Carboxyl-Terminal Hydrolase 27, MRX105	Wainer Katsir and Linial (2019)
4	XIAP	X-linked inhibitor of apoptosis	API3, BIRC4, IAP-3, ILP1, MIHA, XLP2, HIAP-3	Wainer Katsir and Linial (2019)
5	DDX3X	DEAD-BOX helicase 3 X-linked	DBX, DDX14, DDX3, HLP2, CAP-Rf, MRX102	Wainer Katsir and Linial (2019)
6	XIST	X inactive specific transcript	DXS1089, DXS399E, LINC00001, NCRNA00001, SXI1, swd66	Wainer Katsir and Linial (2019)
7	DMD	Dystrophin	BMD, CMD3B, DXS142, DXS164, DXS206, DXS230, DXS239, DXS268, DXS269, DXS270, DXS272, MRX85	Wainer Katsir and Linial (2019)
8	TMSB4X	Thymosin beta 4 X-linked	FX, PTMB4, TB4X, TMSB4	Wainer Katsir and Linial (2019)
9	CSF2RA	Colony stimulating factor 2 receptor subunit alpha	CD116, CDw116, CSF2R, CSF2RAX, CSF2RAY, CSF2RX, CSF2RY, GM-CSF-R-alpha, GMCSFR, GMR, SMDP4	Wainer Katsir and Linial (2019)
10	IL3RA	Interleukin 3 receptor subunit alpha	CD123, IL3R, IL3RAY, IL3RX, IL3RY, hIL-3Ra	Wainer Katsir and Linial (2019)
11	PRKX	Protein KINASE X-linked	PKX1, EC 2.7.11.1, Protein Kinase PKX1	Wainer Katsir and Linial (2019)
12	OTC	Ornithine carbamoyltransferase	OCT29D, OTCase	Wainer Katsir and Linial (2019)
13	IRAK1	interleukin 1 receptor associated kinase 1	IRAK, pelle,	Odhams (2019)
14	TLR7	toll-like receptor 7	TLR7-like	Odhams (2019)
15	CXorf21	Chromosome X open reading frame 21	FLJ11577, Uncharacterized protein CXorf21	Odhams (2019)
16	TMEM187	Transmembrane protein 187	CXorf12, DXS9878E, ITBA1, Protein ITBA1	Odhams (2019)
17	CD40L	CD40 ligand	CD154, CD40L, HIGM1, IGM, IMD3, T-BAM, TNFSF5, TRAP, Gp39, HCD40L	Wang (2016)
18	CXCR3	C-X-C motif chemokine receptor 3	CD182, CD183, CKR-L2, CMKAR3, GPR9, IP10-R, MigR	Wang (2016)

## Cluster of differentiation 40 ligand (CD40L)

CD40L is a type II transmembrane protein which is belonged to tumor necrosis factor (TNF) family of cytokines. It is a 261 amino acids protein with a molecular weight of 35 KDa containing an amino-terminal intracellular domain, a transmembrane domain, and a carboxy-terminal extracellular domain (Laman et al. 1996).

The signaling cascade of the cluster of differentiation 40 (CD40), upon its interaction with CD40L, is initiated by the recruitment of TNF receptor-associated factor (TRAF) adaptor proteins to the cytoplasmic tail of CD40. Various members of the TRAF family, including TRAF-1, TRAF-2, TRAF-3, TRAF-5, and TRAF-6 were detected to associate with the receptor either directly or indirectly. The outcome is the activation of various transcription factors, including nuclear factor kappa-light-chain-enhancer of activated B cells (NF-κB), activator protein 1 (AP-1) and nuclear factor of activated T cells (NFAT) (Schonbeck and Libby 2001).

CD40 engagement on dendritic cells (DCs) by CD40L on CD4+ T cells, increases expression of major histocompatibility complex (MHC) molecules as well as costimulatory signals including, cluster of differentiation 80 (CD80), cluster of differentiation 86 (CD86) and cytokines on DCs. These enhancements which are called licensing, in turn, increase CD4+ T cell responses (Ma and Clark 2009; Abbas et al. 2017). Licensing also renders DCs to induce activation of naïve CD8+ T cells and promote their priming towards effector cytotoxic T lymphocytes (CTLs). This explains the need for CD4+ T cells in the activation and formation of CTL responses (Andrade et al. 2005). By these ways, CD40-CD40L interaction amplifies cellular immune responses exerted by CD4+ and CD8+ T cells. On the other hand, regulatory DCs do not express CD40 despite the presence of stimulatory signals in the cell culture system (Ma and Clark 2009). This subset of DCs produces a great amount of the immunosuppressive cytokines, including interleukin-10 (IL-10) and transforming growth factor-beta (TGF-β) and drives the generation of regulatory T cells (Tregs) (Ma and Clark 2009).

CD40 ligation on B cells influences various aspects of humoral immunity. The ligation of CD40 promotes B cell activation and proliferation. Upregulation of MHC II molecules and costimulatory ligands, CD80 and CD86, was detected on B cells (Karnell et al. 2019). Crucial events which ultimately lead to germinal centers (GCs) formation, are triggered after the interaction between CD40 on B cells and CD40L on T cells. This interaction promotes B cell proliferation in the dark zoon of GCs (Abbas et al. 2017). Moreover, the proliferated cells located in the light zoon of GCs, are induced to express the enzymes, including activation-induced cytidine deaminase and uracil-N glycosylase, required for class switching and affinity maturation process of the antibodies (Abbas et al. 2017). CD40-CD40L interaction is implicated in the development of follicular dendritic cells (FDCs) which are crucial for the maintenance of follicles architecture in the secondary lymphoid organs as well as in the selection events of B cells undergone affinity maturation process (Sullivan 1996). Accordingly, patients with hyper IgM syndrome which are deficient in CD40 or CD40L, demonstrate a defect in GCs formation, accompanied by the impaired formation of FDCs (Sullivan 1996). In summary, CD40 ligation on B cells, contributes to GCs formation in which B cell proliferation and differentiation to the antibody-producing cells as well as isotype switching and affinity maturation of the antibodies are conducted.

Similar to DCs, macrophages also serve as antigen-presenting cells for T cells. Thus, the similar licensing process assumed to DCs, can be attributed to macrophages. This enhances the immunostimulatory activity of macrophages leading to the efficient activation of T cell responses. Accordingly, macrophage CD40 signaling are attributed to the pathogenesis of various human autoimmune disease and animal models of autoimmunity (Suttles and Stout 2009). Moreover, a T cell-independent pathway has been discovered for the activation of macrophages. Andrade et al. detected CD40 stimulated-macrophages infected with an intracellular infection agent, *Toxoplasma Gondi*, were able to destroy the pathogen despite the lack of interferon-gamma (IFN-γ) or reactive nitrogen radicals. The microbicidal effect of these infected macrophages was dependent on Tumor necrosis factor-alpha (TNF-α) suggesting CD40 engagement on macrophages triggers TNF-α production (Andrade et al. 2005). In the following sections, a review of the literature demonstrating participation of CD40-CD40L interaction in the pathogenesis of autoimmune diseases will be presented.

In rheumatoid arthritis (RA), increased number of CD4+ CD40L+ T cells expressing high CD40L transcripts was detected. When the patients were stratified according to the disease activity, a significant correlation was found between CD40L transcript levels and clinical activity of the disease in the patients (Román-Fernández 2019). Ligation of CD40 on synovial tissues of the RA patients, by CD40L+ T cells induced production of TNF-α. These results suggest the activation of CD40 on synovial tissues promotes the generation of the inflammatory microenvironment in the surrounding tissues through the production of proinflammatory cytokines (Harigai 1999). According, the production of TNF-α was detected from monocytes obtained from the synovial tissues of RA patients, upon stimulation with CD40L (Sekine et al. 1998).

A certain allele of CD40 has been identified in association with the higher risk of systemic lupus erythematosus (SLE) in the polymorphism studies (Devi et al. 1998). Accordingly, increased expression of CD40L was observed, at least in some SLE patients. T helper (Th) cells from SLE patients continued to express a high level of CD40L, while CD40L expression in Th cells from the control group was diminished at 24 and 48 h of the stimulation. Moreover, Th cells from SLE patients, induced the higher level of costimulatory molecules in B cells in an in vitro co-culture system (Koshy et al. 1996). Desai-Mehta et al. identified a similar finding. A significant increase of CD40L+ CD4+ T cells in the peripheral blood mononuclear cells (PBMCs) of patients with active lupus was detected. In addition, CD8+ T cells and B cells from these patients showed a de novo expression of CD40L. Utilization of a blocking monoclonal antibody against CD40L impressively inhibited production of the antinuclear autoantibodies in the patients (Desai-Mehta et al. 1996). In the advanced stages of lupus nephritis, increase of CD40 in the parenchymal and non-parenchymal cells of kidney was detected (Yellin 1997). A comprehensive summary of the literature on the CD40L role in SLE has been described by Yazdany et al. (Yazdany and Davis 2004).

Infiltrated CD40L+ T cells were detected in the lesions of multiple sclerosis (MS) patients (Gerritse 1996; Jensen et al. 2001). Analysis of CD40 and CD40L expression in the experimental autoimmune encephalomyelitis (EAE), the animal model of MS, showed upregulation of these molecules during clinical attacks (Gerritse 1996). Monocytes, macrophages and activated microglia were found to be the major cells expressing CD40 in MS patients. Besides, the endothelial cells expressed CD40 either in a constitutive or inducible manner (Wheway et al. 2013; Aarts 2017). CD40 activation of endothelial cells resulted in the expression of adhesion molecules, including E-selectin, vascular cell adhesion protein 1, and intercellular adhesion molecule 1. Accordingly, T cell adhesion to the endothelial cells was increased, facilitating the migration of T cells across the blood–brain barrier into the central nervous system (CNS) (Omari and Dorovini-Zis 2003). Pathologic activation of microglia, resident macrophages of the brain, was shown to be associated with chronic inflammatory diseases, including MS (Luo 2017). It was shown that microglia stimulated with CD40L generate an inflammatory milieu in the brain through upregulation of TNF-α, interleukin-12 (IL-12), nitric oxide, matrix metalloproteinase 9 (MMP9) and monocyte chemoattractant protein 1 and C-X-C motif chemokine 10 (Aarts 2017; D'Aversa et al. 2002). A comprehensive review of the implication of CD40-CD40L axis in MS and animal models of the disease has been performed by Aarts et al. (2017). Lamina propria T cells isolated from the patients with inflammatory bowel diseases (IBDs), including Crohn's disease and ulcerative colitis, expressed a high level of CD40L molecules and were able to enhance the production of IL-12 and TNF-α from monocytes. Activated T cells from IBDs patients also produced a prolonged expression of CD40L. Moreover, a higher number of CD40 + cells, including macrophages and B cells, was found in the inflamed mucosa of the patients compared to the healthy control individuals (Liu et al. 1999). Besides macrophages and B cells, endothelial cells from the lamina propria vasculature upregulate CD40, facilitating infiltration of CD40L expressing T cells into the tissues (Danese et al. 2006). The higher percentage of CD40+ cells in the colonic mucosa of IBDs patients found to be directly associated with the disease activity (Polese 2002).

Analysis of naïve T cells and B cells from healthy women showed biallelically expressed CD40L gene at least in some cells compared to healthy men lymphocytes. Female B cells of SLE patients also exhibited biallelic expression of CD40L gene as well as increased expression level providing evidence in reactivation of immune-related gene CD40L in SLE female lymphocytes and consequent enhanced immune response in the female patients (Wang 2016). Consistently, stimulated PBMCs from healthy females and males with Klinefelter syndrome, men with XXY karyotype, showed higher CD40L expression level in T cells and higher number of Cluster of differentiation 3 (CD3)+ CD40L+ T cells compared with healthy males and women with Turner syndrome, women with X karyotype (Sarmiento et al. 2019). Coz et al. identified a CD40L gene duplication in CD4+ T cells of a six-month-old male showing autoimmune disease, resulted in a twice CD40L gene transcripts than that of his unaffected relatives. These CD4+ T cells derived more expression level of activation-induced cytidine deaminase **(**AIDCA) in B cells resulting in more antibody class-switching and lower level of IgM antibody. Interestingly, CD4+ T cells of his mother which showed epigenetic silencing of the duplicated X chromosome, intermediately induced AIDCA expression and antibody class-switching than those of female healthy controls and her son (Coz 2018). A higher level of IgG antibody was also recognized in female collagen-induced arthritis model of rheumatoid arthritis compared to male animal model of the disease by Dimitrijevice et al. Accordingly, a higher frequency of CD40L+ T cells and CD40+ B cells were identified in female lymph nodes of the animals compared to male counterparts (Dimitrijević 2020). Demethylation and increased expression of CD40L gene on the inactive X chromosome in female CD4+ T cells than male lymphocytes was also reported in patients with SLE (Lu et al. 2007). Altogether, these studies point to the contribution of CD40L gene in female bias of autoimmunity.

## Cluster of differentiation 99 (CD99)

CD99 is a highly O-glycosylated transmembrane protein (type 1) which is not belonged to any of the known protein superfamilies. It exerts a fundamental function in the transendothelial migration of leukocytes known as diapedesis (Sullivan and Muller 2014). A hemophilic interaction between CD99 molecules located on monocytes, neutrophils and lymphocytes with those of the endothelial cells, triggers a signaling cascade allowing migration of leukocytes towards the tissues across the tight junctions of endothelial cells (Sullivan and Muller 2014; Schenkel et al. 2002; Dufour et al. 2008; Bixel 2004). A detail of the molecular mechanisms mediating diapedesis was given by Hichey el al (Hickey 2015). CD99 also implicated in the transmigration of DCs from peripheral tissues into the secondary lymphoid organs, where they present the captured antigens to the naive T cells and activate or enhance T cell responses (Torzicky 2012). Moreover, CD99 was identified for its function in the firm adhesion of resting and memory T cells to the vascular endothelium through increasing affinity/avidity of α4β1 integrins. Firm adhesion is a central step in the leukocyte migration from blood into the tissues mediated through the integrins with the high-affinity binding state (Bernard 2000). CD99 activity in increasing affinity/avidity of the integrins, may be redundant or strengthen the similar effect exerted by the chemokines, resulting in the conversion of an integrin from a bent shape (low-affinity integrin) into the extended shape (high-affinity integrin) (Abbas et al. 2017).

In addition to the role in the transendothelial migration of leukocytes, CD99 influenced cytokine production and antigen presentation. Ligation of CD99 on the activated T cells by a CD99 recombinant protein, increased production of proinflammatory cytokines, including interleukin-6 (IL-6) and TNF-α. Engagement of CD99 on monocytes and natural killer cells also resulted in the increased production of IL-6 and TNF-α (Takheaw et al. 2019). Waclavicek et al. demonstrated resting T cells in response to CD99 stimulation and T cell receptor (TCR) activation, proliferate and produce IFN-γ and TNF-α, (Waclavicek 1998). A similar stimulatory role of CD99 for CD4+ T cells was reported by the others (Wingett et al. 1999; Oh 2007). Furthermore, a CD99 requirement for the surface expression of MHC class I (MHC I) has been reported. Given that the extent of CD8+ T cell activation is dependent on MHC I densities presented on the cell surface of antigen presenting cells (APCs), the surface level of MHC I molecules is tightly regulated. Assuming CD99 acts as a mechanism for regulation of the surface MHC I densities, therefore, any CD99 perturbation may affect the expression level of MHC I and consequent CD8+ T cell activation (Brémond 2009; Sohn, et al. 2001). T cell activation by the above mentioned mechanisms, contributes to the pathogenesis of autoimmune diseases being promoted mainly through T cell immune responses (Dardalhon et al. 2008; Skapenko et al. 2005).

On the contrary to the immunostimulatory functions of CD99, ligation of CD99 on plasma cells (PCs) interfered with the chemotactic translocation of PCs toward C-X-C motif chemokine 12 (CXCL12) chemokine (Gil 2015). CXCL12 directs migration of long-lived PCs towards the bone marrow, where the signals from the stromal cells provide the necessary microenvironment for the long survival of PCs (Abbas et al. 2017). Therefore, a high level of CD99 may be in favor of the antibody-mediated autoimmune diseases (Mahévas et al. 2013), as a reduction in the number of autoreactive long-lived PCs is expected. However, additional studies are necessary to address this issue.

Other studies unrevealed a possible etiology of the contradictory roles of CD99 (Cernadas et al. 2009; Alberti 2002). Cernadas et al. identified a regulatory role for CD99 in the expression of cluster of differentiation 1a (CD1a) in which isoform variants of CD99 possess opposing effects on the CD1a expression in human monocyte-derived DCs. While CD99 long-form decreased CD1a transcription, the short-form of CD99 counteracted this effect (Mahiddine 2016). Basically, CD1a molecules which are structurally similar to MHC I, are known for their functions in the presentation of lipid and glycolipid antigens to gamma delta T cells. DCs expressing CD1a produces a great amount of IL-12 and promotes T helper 1 (Th1) responses in favor of some autoimmune diseases (Cernadas et al. 2009). Therefore, CD1a acquisition in DCs may be accompanied with obtaining a functional trait and CD99 regulates DCs function through controlling CD1a expression. As a conclusion, different isoforms of CD99 molecules may be responsible for the various effects exerted on the immune responses by CD99.

In psoriasis, the disease progression was accompanied by the elevated CD99 protein in the dermis and epidermis. On the other hand, a decrease of CD99 protein in both dermis and epidermis, was observed in the remission phase of the disease (Belonogov and Ruksha 2013). An increase in the CD99 expression level in the PBMCs and inflamed mucosa tissues obtained from the patients with IBDs was reported Zhou et al. The level of CD99 molecules was positively associated with the disease activity. These results suggested CD99 may play a pathologic role in the disease course of IBD and serves as an indicator of the disease activity in the patients (Zhou et al. 2017). CD99 inhibition in the animal model of MS, EAE, reduced leukocytes infiltration, including CD4+ T cells, CD8+ T cells and B cells into the CNS and attenuated the disease in the animals (Winger et al. 2016). In conclusion, despite the divergent effects of CD99 on the immune responses, the insights coming from the human autoimmune diseases and animal models of autoimmune diseases, support the pathologic roles of CD99 in autoimmunity. In addition, further experiments are required to evaluate contribution of CD99 in the female bias of autoimmunity.

## Lysosome-associated membrane protein 2 (LAMP-2)

LAMP-2 is a highly glycosylated type I transmembrane protein with a molecular weight of about 40–45 kDa for the protein backbone and a total molecular weight of approximately 120 kDa for its glycosylated protein. Both LAMP-2 and LAMP-1 constitute around 50% of the lysosome membrane proteins. They were proposed to be responsible for keeping the lysosomal membrane integrity against the hydrolytic enzymes located whiten the lysosome lumen. The overall structure of LAMP-2 contains an amino-terminal lumenal domain, a transmembrane domain and a carboxy-terminal cytosolic domain.

Alternative splicing of the primary messenger ribonucleic acid (mRNA) of LAMP-2 generates various isoforms, including LAMP-2a, LAMP-2b, and LAMP-2c which are different in the amino acid sequences of the transmembrane and cytosolic domains (Eskelinen 2006). It was shown that LAMP-2a isoform is different in its structural and functional properties from the other isoforms. In this regard, LAMP-2a involves in the autophagy process mediated by chaperons, called Chaperon mediated autophagy (CMA). In CMA, misfolded cytoplasmic proteins are recognized and targeted by the chaperons for the degradation in lysosomes (Cuervo and Dice 2000; Kaushik and Cuervo 2012). Herein, 70 KDa heat shock cognate proteins (Hsc70) recognize the cytoplasmic proteins containing the amino acid sequence of KFERQ and deliver them to the lysosomal membrane receptor, LAMP-2a. After binding of the substrate proteins to LAMP-2a, LAMP-2a is multimerized, facilitating unfolding of the substrate proteins and their translocation into the lysosomal lumen. The modulation of the LAMP-2a level is a way by which the cells regulate the extent of CMA (Kaushik and Cuervo 2012).

Antigen presentation is a vital process for the activation of T lymphocytes in which a protein (or autoantigen) is degraded, and the resulting linear epitopes bind to MHC molecules and presented on the surface of APCs to be recognized by T cells (or autoreactive T cells). Classically, it is well known the endogenous cytoplasmic proteins are loaded on MHC I molecules and present to CD8+ T cells, while the exogenous proteins are loaded on MHC class II (MHC II) molecules and present to CD4+ T cells. The degradation of the endogenous and exogenous proteins is performed in the proteasomes and lysosomes, respectively (Abbas et al. 2017).

Autophagy enhances presentation of the endogenous antigen on MHC I and activates CD8+ T cells activation. Moreover, it was recognized that the endogenous proteins (cytosolic autoantigens) derived from autophagosomes can be loaded on MHC II and activate CD4+ T cells (Zhou 2005). These findings highlight the possible roles of LAMP-2a in the antigen presentation to autoreactive T cells through CMA leading to the initiation or enhancement of immune responses against autoantigens. An impairment in the exogenous antigen presentation on MHC II molecules in the LAMP-2 deficient B cells was also reported, providing a direct evidence for the contribution of LAMP-2 in priming and activation of T cells against the antigens (Crotzer et al. 2010), however, it remains to be discovered whether LAMP-2 contribution in the antigen presentation to T cell is mediated through regulation of the autophagy machinery or by a direct influence on the components involved in the pathway of antigen presentation on MHC molecules.

In addition to affecting antigen presentation, autophagy is implicated in the activation of NF-κB. NF-κB is a central transcription factor in the transcription of the genes involved in innate and adaptive immune responses such as proinflammatory mediators, adhesion molecules and chemokines (Liu et al. 2017). Dysregulation of the NF-κB pathway has been demonstrated in many autoimmune diseases and the pathway components have been considered as the potential therapeutic targets in inflammatory and autoimmune diseases (Tak and Firestein 2001; Herrington et al. 2015; Bacher and Schmitz 2004). Criollo et al. uncovered autophagy is needed for NF-κB activation. They showed stimulation of autophagy removes the inhibitory effect of inhibitor of NF-κB which acts as the inhibitor of NF-κB activation (IκB). Therefore, NF-κB is activated and migrate into the nucleus, where it upregulates expression of the genes involved in the inflammatory response (Criollo 2010). Accordingly, Kanayama et al. showed the enhanced activation of NF-κB in macrophages by autophagy. Activation of autophagy led to the degradation of A20 which negatively regulates NF-κB gene expression (Heyninck 1999; Kanayama 2015).

Autophagy also affects T cell proliferation, survival and differentiation. Deficient T cells in the autophagy genes show impaired proliferation and increased level of apoptosis. Khan et al. observed that costimulation of nucleotide-binding oligomerization domain-containing protein 2 and toll-like receptor 4 in DCs induced autophagy genes and increased production of the cytokines, including IL-6, IL-12 and IFN-γ and costimulatory molecules, CD40, CD80 and CD86, in T cells. These results suggested autophagy participates in the induction of Th1 responses (Khan 2016). In another study, the role of autophagy in the induction of both IFN-γ and interleukin-17 (IL-17) cytokines and inhibition of T helper 2 (Th2) responses was identified (Reed et al. 2013). In the concept of autoimmunity, autophagy may increase the proliferation and survival of autoreactive T cells. In addition, it increases the differentiation of Th1 and Th17 which are implicated in the pathogenesis of many autoimmune diseases (Dardalhon et al. 2008; Skapenko et al. 2005). The humoral immune response is also influenced by autophagy. It was shown that autophagy participates in B cell survival during developmental stages (Miller 2008). In addition, it is required for the antibody production by plasma cells, maintenance of plasma cell and long-lived humoral immunity (Pengo 2013).

The above mentioned mechanisms may somewhat explain the contribution of autophagy in the pathogenesis of some autoimmune diseases. Clark et al. found activation of autophagy in B cells in various developmental stages in a lupus mouse model and the human disease (Clarke 2015). Accordingly, the levels of autophagic transcripts were positively associated with the disease activity and anti-double stranded DNA antibodies (anti-dsDNA) levels in the PBMCs of SLE patients (Wu 2017). Next studies should address contribution of LAMP-2 in the female bias of SLE.

In RA, a higher expression level of autophagy genes in the synovial tissues of the patients was observed. A direct correlation between these transcripts levels and the serum features of disease activity, including C-reactive protein, erythrocyte sedimentation rate, anti-cyclic citrullinated peptide antibodies and rheumatoid factor was also observed (Zhu 2017). Accordingly, Loosdregt et al. observed the enhancement of autophagy in CD4+ T cells from RA patients. Inhibition of the autophagy in the mouse model of RA, reduced disease incidence and severity (Loosdregt 2016).

The disease severity in the mouse model and human disease of MS was associated with the transcriptional and protein levels of an autophagy gene, autophagy-related gene 5 (Alirezaei 2009). Another evidence in the participation of autophagy in MS, comes from the ameliorative effect of rapamycin, an inhibitor of autophagy, in the relapsing–remitting EAE (Esposito 2010).

Besides immunoenhancing effects of autophagy, it also negatively affects the immune responses through interfering with NF-κB activation, maturation and migration of DCs and activation of Tregs (Ghislat and Lawrence 2018; Paul et al. 2012; Zang 2016). These may explain the reported protective roles of autophagy in autoimmunity (Bhattacharya et al. 2014). A comprehensive review of the literature on the implication of autophagy in autoimmunity, was discussed by Yin et al. and Yang et al. (Yin, et al. 2018; Yang et al. 2015a).

## Interleukin-1 receptor-associated kinase-1 (IRAK-1)

IRAK-1 is a serine-threonine kinase involved in the signaling pathway of toll-like receptors (TLRs) and interleukin-1 (IL-1) receptors. It consists of (1) an amino-terminal domain designated death domain which mediates IRAK-1 interaction with IRAK-4 which is another serine-threonine kinase associated to the TLR and IL-1 receptors (2) a carboxy-terminal domain which mediates IRAK-1 interaction with TRAF-6 (3) a kinase domain which mediates kinase activity of IRAK-1 resulting in the phosphorylation and activation of TRAF-6 (4) a proline–serine–threonine-rich domain (Hynes and Nair 2014; Rhyasen and Starczynowski 2015).

In the signaling pathways of TLRs and IL-1 receptors, engagement of the receptors with their ligands, including TLR ligands and cytokines, activates a number of the downstream adaptor proteins and kinase proteins resulting in the activation of NF-κB transcription factor. The signaling cascade is initiated by the recruitment of myeloid differentiation primary response 88 (MyD88) to the cytoplasmic toll/IL 1 receptor domain of the receptors, followed by the association of IRAK-4 to the receptor. Thereafter, IRAK-1 is recruited and activated, leading to the phosphorylation and activation of TRAF-6. TRAF-6, in turn, activates IκB kinase (IKK) complex. This triggers dissociation of the IκB complex from NF-κB, followed by the NF-κB activation and its translocation to the nucleus, where it upregulates transcription of the genes involved in the activation of immune responses (Cooke et al. 2001).

Activation of NF-κB in macrophages, initiates transcription of various genes cooperating to elicit and amplify inflammation including pro-inflammatory cytokines, TNF-α, interleukin-1 beta (IL-1β), IL-6 and IL-12, as well as lipid mediators, prostaglandins and leukotrienes and chemokines. (Abbas et al. 2017).

Inhibition of NF-κB activation prevented DCs maturation through downregulation of MHC molecules and costimulatory proteins (Rescigno et al. 1998; Hernandez 2007; Albrecht et al. 2008). NF-κB activation in T cells also modulates immune responses through affecting differentiation of naïve T cells towards Th1, Th2 and Th17 (Aronica, et al. 1999; Li-Weber et al. 2004; Ruan 2011). A valuable detail of the literature for NF-κB roles in T cell differentiation was given by Oh et al. (Oh and Ghosh 2013). As a conclusion, IRAK-1 contribute to autoimmunity through promoting differentiation and activation of various T cells subsets.

On contrary, NF-κB was reported to maintain tolerance and prevent autoimmunity. It participates in the development of natural Tregs in the thymus and supports the maintenance and functions of Tregs (Oh and Ghosh 2013; Sun et al. 2013). Besides, by controlling the functional maturation of DCs, NF-κB keeps DCs in the tolerogenic state and prevents activation of autoreactive T cells (Dissanayake 2011).

Several researchers documented the epigenetic alteration of the target genes by IRAK-1. Differential methylation of 245 CpG sites in DNA of the stimulated T cells from SLE patients in comparison with the healthy individuals was reported which was mostly concentrated in MHC and interferon response l loci (Woodman 2013). Accordingly, Koelsch et al. revealed methyl CpG binding protein 2/IRAK-1 loci affected genetic loci of MHC and interferon-regulated genes in the SLE patients through several epigenetic mechanisms, including DNA methylation, microRNA expression and histone modifications (Koelsch 2013).

Polymorphism studies revealed some allelic variants of IRAK-1 are associated with the higher risk of autoimmunity (Li 2015). Certain allelic variants of IRAK-1 were associated with the increased risk of autoimmune thyroid diseases, including Hashimoto's thyroiditis and Graves' disease (Song 2015). rs3027898 C/A variant allele of IRAK-1 was found to be associated with the susceptibility to RA (Zhang 2013). IRAK-1 can contribute to the pathogenesis of RA through modulation of tissue remodeling enzymes. It was demonstrated IRAK-1 induces matrix metalloproteinase 13 (MMP13) generation from human chondrocytes in response to IL-1 stimulation (Ahmad et al. 2007). MMP13 degrades collagen and aggrecan and contributes to the cartilage destruction in RA (Burrage et al. 2006).

IRAK-1 gene was identified as a crucial risk gene in the pathogenesis of SLE (Jacob 2009; Dieudé 2011). Besides, polymorphisms of another gene encoding A20-binding inhibitor of NF-κB1 (ABIN1) also predispose to SLE and other autoimmune diseases. ABIN1 which is a polyubiquitin binding protein, acts as an inhibitor of NF-κB signaling pathway. Prevention of ABIN1 polyubiquitin binding resulted in the activation of NF-κB and consequent inflammatory response (G'Sell et al. 2015). Accordingly, the genetically modified mice expressing a mutant of ABIN1 in which polyubiquitin binding region of the protein was defective, generated an autoimmune disease similar to the lupus nephritis. Suppression of IRAK-1 in these animals prevented lupus nephritis suggesting IRAK-1 is a downstream target of the regulatory action of ABIN1 in the NF-κB signaling pathway and perturbation of IRAK-1 expression may counteract the inhibitory action of ABIN1 in NF-κB activation (Nanda et al. 2016). Upregulation of IRAK-1 in both transcriptional and protein levels was detected in SLE patients. Inhibition of IRAK-1 in naïve T cells from the SLE patients prevented Th17 differentiation. Therefore, IRAK-1 may contribute to the SLE pathogenesis through expanding Th17 responses (Zhou 2018). Chiang et al. provided evidence elucidating another mechanism of IRAK-1 contribution to the SLE pathogenesis. They found inhibition of IRAK-1 (and IRAK-4) differentially influenced activation of various immune cells in response to TLR7 or TLR9 stimulation. IRAK1 inhibition prevented plasmacytoid dendritic cells (pDCs) activation, not the other human immune cells (Chiang et al. 2011). It is known activation of pDCs by self DNA and RNA, triggers production of IFN-α which is implicated in the SLE pathogenesis (Sisirak 2014). Therefore, IRAK1 may be involved in the SLE pathogenesis by activating pDCs. Elevated serum level of IFN-α in New Zealand hybrid mouse model of SLE grafted with female hematopoietic cells rather than the male hematopoietic cells, points to the involvement of IFN-α in female bias of SLE. As IRAK-1 gene is involved in the signaling pathway downstream of IFN-α, IRAK-1 gene may be considered as an essential factor in the female-dependency of SLE (David 2014). In another study, it was exhibited that female SLE T cells increased expression of IRAK1 and showed altered X chromosome inactivation pattern compared to the male SLE patients, while female healthy controls showed a similar expression profile of IRAK-1 to those of male SLE patients indicating a sex-biased role of IRAK-1 in SLE disorder (Syrett et al. 2019).

A female-biased contribution of IRAK-1 in the pathogeneses of experimental colitis, the experimental animal model of IBDs, was observed by Berglund et al. The male IRAK-1 deficient mice showed significant protection against the disease compared to the female IRAK-1 deficient mice (Berglund 2009). IRAK-1 was shown to be vital for returning of the activated T cells into the inflamed intestine, after their priming in the peripheral lymphoid organs (Heiseke et al. 2015). It has been exhibited the therapeutic intervention such as vesicles containing miR-146a, attenuate the experimental colitis in the animals by inhibition of TRAF-6 and IRAK-1 (Wu 2019).

The possible attribution of IRAK-1 in the pathogenesis of scleroderma, comes from the study in which inherited IRAK-1 deficiency in a boy resulted in the poor responses of his fibroblasts to the TLRs agonist (Della Mina 2017). TLRs stimulation by their corresponding ligands or agonists participate in the disease pathogenesis through the activation of fibroblasts which promotes fibrosis of the surrounding tissues, the dominant characteristic observed in scleroderma patients (Bhattacharyya et al. 2017).

## Toll-like receptor 7 (TLR7)

TLR7 is an intracellular TLR located in the endosomes and lysosomes and binds single-stranded RNA (ssRNA). Moreover, it recognizes guanosine and oligoribonucleotides containing uridine, which are the resulting products of ssRNA degradation in ribosomes (Miyake 2018; Fujiwara et al. 2017; Shibata 2016; Sarvestani 2014). Initial binding to the oligoribonucleotides is a prerequisite for guanosine binding (Miyake 2018; Shibata 2016). This dual binding may synergistically enhance activation of TLR7 (Miyake 2018; Shibata 2016). The crystallography of TLR7 represented a dimerized m-shaped structure for the receptor, containing two ligand-binding sites. The first site bound guanosine, and the second site was responsible for the recognition of uridine moieties in ssRNA and oligoribonucleotides (Zhang 2016).

Upon binding to the ligand, TLR7 activates and stimulates the production of proinflammatory cytokines and chemokines, including TNF-a, IL-6, IL-12 and regulated upon activation, normal T cell expressed and secrete chemokine (C-C motif) ligand 5 (CCL5), a chemokine which acts a chemotactic factor for T cells, eosinophil and basophil (Bishara et al. 2012). The immunostimulatory functions of TLR7 are mediated through interferon regulatory factor 5, NF-κB and AP-1 transcription factors (Shevlin and Miggin 2014). Moreover, TLR7 activation triggers the production of type I IFNs via interferon regulatory factor 7 (IRF7) transcription factor. After TLR7 activation, the receptor traffics from the endosomes and lysosomes towards the peripheral regions of the cell. ADP ribosylation factor like GTPase 8b (Arl8b) and pleckstrin homology domain-containing family M member 2 (Plekhm2) deficient pDCs are impaired in the TLR7 trafficking suggesting Arl8b and Plekhm2 are the essential components of TLR7 trafficking machinery. In addition, a perturbation in type I IFNs production in the Arl8b and Plekhm2 deficient pDCs was observed indicating trafficking of TLR7 to the cell periphery is required for the generation of type I IFNs by pDCs. Mechanistically, these proteins mediate cytoskeletal changes required for the organelle translocations across the cell. This allows for the movement of TLR7 vesicles towards the periphery, where the IRF7 transcription factors are located. Afterwards, IRF7 are activated and enhance transcription of type I IFNs. The etiologic role of type I IFNs particularly interferon-alpha (IFN-α) in the pathogenesis of SLE has been well-known (Rönnblom and Alm 2001). In a study IFN-α was introduced as a new biomarker of the disease activity in SLE patients (Obermoser and Pascual 2010). Interestingly, a higher IFN-α production was detected in the females than the males after TLR7 stimulation, highlighting the role of TLR7 in the female-biased prevalence of SLE in women (Berghöfer, et al. 2006).

Self nucleic acids which activate TLR7, are delivered to the endosomal or lysosomal compartments through several ways, including (Celhar et al. 2012): (1) Endocytosis of the immune complexes containing self nucleic acids and antibody after binding to Fc gamma receptors (FcγR) of the immune cells (2) Uptake of the complexes containing self nucleic acids and LL-37, a cationic antimicrobial inflammatory peptide which has been identified to facilitate the uptake of DNA and RNA into the cytoplasm (Pandey et al. 2014) (3) Uptake of the complexes containing self nucleic acids and high mobility group box 1, a chromatin-binding protein which is released upon the cell death and serves as a damage-associated molecular pattern molecule eliciting the immune responses (Yang et al. 2015b) (4) Uptake of neutrophil extracellular traps, the structures containing DNA, histones and granule proteins of neutrophils released from the cell upon its activation and contributes to the microbial killing (Li and Tablin 2018) (5) Uptake of apoptotic cell debris containing self-nucleic acids (Celhar et al. 2012).

Recently, age-associated B cells (ABCs) have been discovered, which contribute to the autoimmunity. This subset of B cells expressed T-box protein expressed in T cells transcription factor and accumulated with age (Rubtsova et al. 2015). TLR7 signaling was identified to be crucial for the autoantibody production by the ABCs and the cell accumulation in the inflamed organs (Rubtsov et al. 2013). Late apoptotic cell debris has been implicated in the production of autoantibodies in SLE (Jung and Suh 2015). A prerequisite for TLR7 in the autoantibody production, including anti-dsDNA and anti-histone antibodies, was documented in the animal model of SLE after administration of the late apoptotic cell debris. TLR7 also promoted complement component 3 deposition in the glomerulus. These findings point to the pathologic role of TLR7 in the autoantibody production (Pan 2010). Moreover, robust activation of TLR7 generated and expanded spontaneous GCs in SLE through upregulation of cyclic D3 (CCND3) (Fan et al. 2018; Ren 2016). CCND3 contributes to the formation and expansion of GCs and its upregulation in SLE patients is associated with the presence of the remained apoptotic cells. The remained apoptotic cells are resulted from the impairment of the apoptotic cell death and decreased clearance of the apoptotic cells in SLE patients. The rupture in the plasma membrane of the remained apoptotic cells releases intracellular antigens such as self DNA and RNA (Colonna et al. 2014). Binding of the immune complexes containing self nucleic acid (self DNA and RNA) and autoantibody to the B cell receptors (BCR), triggers endocytosis and subsequent delivery of the self nucleic acid to the endosomal compartments resulting in the activation of TLR7 signaling. As a result, the autoreactive B cells are activated and increase the expression of CCND3. CCND3, in turn, vigorously promotes B cells proliferation and GCs formation. In GCs, B cells actively continue to the production of autoantibodies (Fan et al. 2018; Soni, et al. 2014). Enhanced TLR7 expression in B cells increases B cells sensitivity, polyclonal proliferation and differentiation into the antibody-producing cells (Bekeredjian-Ding, et al. 2005).

The high expression level of TLR7 in transient B cells renders them hyper-responsive to the BCR stimulant and TLR7 stimulation, which triggers production of a great amount of the antibodies, including anti-RNA antibodies. Transient B cells are immature B cells migrated from the bone marrow into the spleen and ultimately develop to follicular B cells, the main subset of B cells which are involved in the humoral immune response against T dependent antigens (Loder 1999). These findings imply high TLR7 expression in the developmental stages of B cells renders them to an autoreactive phenotype (Giltiay 2013).

Distinct expressions of TLR7 and TLR9 in SLE patients were associated with the distinct autoantibody patterns. The levels of autoantibodies, including anti-extractable nuclear antigens and anti-dsDNA antibodies, were correlated with TLR7 and TLR9 levels, respectively suggesting the TLRs effect in directing the autoantibody profiles of SLE patients (Chauhan et al. 2013). TLR7 activation in neutrophils also influences immune complex-mediated autoimmune diseases such as SLE (Toong et al. 2011), as it triggered the breakdown of FcγRIIA (CD32) located on pDCs and monocytes. Given the involvement of FcγRIIA in the phagocytosis and clearance of immune complexes, FcγRIIA degradation interferes with the immune complex clearance (Lood et al. 2017).

Besides affecting humoral immune responses, TLR7 also influences T cell responses in autoimmunity. TLR7 activation increased production of IL-12 from macrophages of the patients with immune thrombocytopenia and promoted Th1 polarization resulting in the reduction of the platelet count (Yang 2011; Spranger 2010). Accordingly, TLR7 deficient mice developed Th2 responses against influenza A virus, suggesting TLR7 inhibits Th2 responses. Simultaneously, an accumulation of myeloid-derived suppressor cells, the immunosuppressive cells of immune system with the myeloid origin, was found in the TLR7 deficient mice (Jeisy-Scott 2011). TLR7 activation also participates in Th17 differentiation and functions in pDCs (Yu, et al. 2010).

CD8+ T cells purified from the human immunodeficiency virus-1-infected patients significantly activated in response to the TLR7 engagement, as evidenced by the expression of the activation markers and IFN-γ production by CD8+ T cells, suggesting TLR7 activation in CD8+ T cells triggers their activation (Song 2009). In addition, an enhanced cross-presentation of the cell-associated antigens to CD8+ T cells was observed using influenza A-infected cell lines in comparison with the uninfected cell lines, resulting in the dramatic expansion and activation of CD8+ T cells. The increase in the cross-priming of CD8+ T cells was dependent on TLR7 recognition of influenza A virus RNA (Wei et al. 2010). Expanding these findings to the autoantigens, viral infection can trigger the onset or progression of autoimmune diseases, as the virus-infected DCs is more potent in the cross-priming of autoreactive CD8+ T cells due to the immunostimulatory effects of viral RNA in TLR7 activation (Getts et al. 2013). Altogether, these studies open the insights to the TLR7 functions in the activation of the cellular immune responses (both CD4+ T cells and CD8+ T cells) and possible roles in the onset or promotion of autoimmune diseases.

Ligation of TLR7 in DCs was accompanied by the upregulation of CD80, CD86, C-C chemokine receptor type 7 and CD40 molecules and production of IL-6 and IL-12, highlighting the role of TLR7 in DCs maturation and promotion of Th1 immune responses (Larangé et al. 2009). TLR7 activation of DCs also promoted autoreactive Th17 responses in the experimental autoimmune uveitis mice, leading to the exacerbation of the diseases (Xiao 2016). DCs stimulated with TLR7 ligands generated B -cell activating factor which is a major cytokine in the proliferation, differentiation and survival of B cell and enhanced activation of autoreactive B cell and production of autoantibodies (Yu 2011).

Stimulated PBMCs from females and Klinefelter syndrome patients exhibited significantly higher expression level of TLR7 compared to PBMCs from male and Turner syndrome patients (Sarmiento et al. 2019). Consistently, Souyris et al. demonstrated biallelic TLR7 gene in naïve B lymphocytes, monocytes, and plasmacytoid dendritic cells from healthy women and men with Klinefelter syndrome as well as a greater expression level of TLR7*.* Besides, these TLR7-stimulated biallelic B cells produced more than two-fold of IgG antibody than monoallelic B cells (Souyris, et al. 2018). Several studies demonstrated the contribution of TLR7 copy numbers to the female bias observed in autoimmunity, particularly in SLE (Sarmiento et al. 2019; Yu, et al. 2010; Liao 2014; García-Ortiz 2010; Heintze 2018). Interestingly, a functional polymorphism in the TLR7 gene resulting in the elevated TLR7 expression, predisposed the male gender to SLE (Shen 2010) The Y-linked autoimmune accelerating (Yaa) locus which is located on the Y chromosome and contains a cluster of the X-linked genes translocated into the Y chromosome, confers susceptibility to the autoimmune disease in male mice. TLR7 gene was identified in the Yaa locus and played a major role in the autoimmune phenotypes of these mice manifested as the splenomegaly and glomerular nephritis accompanied with the perturbation of immune responses and leukocytes infiltration into the kidney (Fairhurst 2008; Pisitkun, et al. 2006). In a recent review by Souyris et al., contribution of TLR7 in female bias of autoimmunity has been comprehensively summarized (Souyris et al. 2019).

TLR7 was shown to be involved in the exacerbation of EAE. The protection observed in the TLR7 deficient mice was manifested as the reduced level of autoantibodies, reduced activation of autoreactive T cells and elevation in Foxp3+ Tregs (Lalive 2014). In RA, a strong correlation between TLR7 expression in monocytes and proinflammatory cytokines level, TNF-α, was described (Chamberlain 2013).

In contrast to the stimulatory functions of TLR7 in immune responses, its inhibitory effects were also reported. Dominguez-Villar et al. showed TLR7 ligation induces an anergic state in CD4+ T cells through activation of NFAT transcription factor. This may explain significant dysfunction of CD4+ T cells observed during a chronic viral infection such as HIV, as genomic RNA of the virus constantly triggers TLR7 signaling (Dominguez-Villar et al. 2015; Lederman 2015). TLR7 signaling also increased the suppressive activities of CD4+ CD25+ Tregs which was a result of the increased sensitivity of Tregs to interleukin-2 (IL-2) activation, as IL-2 increases the survival and suppressive activities of Tregs (Forward et al. 2010). These immunosuppressive activities of TLR7 may explain the attenuation of the disease in the EAE model, after treatment of the animals with the TLR7 agonist (O'Brien et al. 2010).

## Ubiquitin carboxyl-terminal hydrolase 27 (USP27X)

USP27X is a deubiquitinating enzyme consists of 438 amino acids and contains a conserved catalytic domain. It removes ubiquitin from the proteins (Guo et al. 2019). Ubiquitin is a small molecule (76 amino acids) which is added to a protein during a process called ubiquitination and marks the proteins for the degradation in proteasomes (Gong et al. 2016). Dysregulation of USP27X was related to some cancers (Lambies 2019; Weber 2016). Given that the possible implication of USP27X in the autoimmunity has not been addressed in any studies, however, a hypothetical implication of USP27X in the autoimmunity can be deduced from the other studies.

USP27X removes ubiquitin from cyclic guanosine monophosphate (GMP)-adenosine monophosphate (AMP) synthase (cGAS) and decreases its turnover. cGAS recognizes cytosolic DNA and subsequently triggers inflammatory response. After DNA sensing, cGAS promotes an enzymatic reaction resulting in the generation of cyclic GMP-AMP (cGAMP). cGAMP binds to the stimulator of interferon genes (STING) protein. Activated STING triggers phosphorylation of TANK binding protein 1 (TBK1). Consequently, phosphorylated TBK1 activates interferon regulatory factor 3 (IRF3), a transcription factor that is involved in the transcription of inflammatory genes particularly type I IFNs (Guo et al. 2019). Macrophages obtained from USP27X deficient mice showed impaired innate antiviral responses against Herpes simplex virus type 1 infection. Mechanistically, a reduction in the interferon-beta (IFN-β) production was observed in the macrophages accompanied with the decrease in cGAMP production, TBK1 phosphorylation and IRF3 activation (Guo et al. 2019). The etiological role of type I IFNs, including IFN-β, in the pathogenesis of autoimmune diseases is well-known documented (Rönnblom and Alm 2001). Accordingly, cGAS activation by self DNA was found to contribute to the etiopathology of autoimmune diseases (Gao 2015).

USP27X may also contribute to the autoimmunity through increasing cell death by apoptosis. Increased apoptosis has been implicated in the pathogenesis of a number of autoimmune diseases such as SLE (Munoz 2008). Pro-apoptotic effects of USP27X are mediated through its binding to phosphorylated and ubiquitinated Bcl-2-like protein 11 proteins, also designated Bim, and dissociation of ubiquitin molecules from this protein (Weber 2016). Bim is a pro-apoptotic protein which upon activation induces apoptosis. Regulation of the Bim level may be mediated either in the expression level or by its degradation in the proteasome after ubiquitination (Sionov et al. 2015). Deubiquitinating enzyme USP27X stabilizes Bim molecules by the removal of ubiquitin molecules. Accordingly, over expression of USP27X induces a pro-apoptotic activity in some of tumor cell lines, including melanoma and non-small cell lung cancer cells (Weber 2016).

It was identified USP27X activates fibroblasts through the deubiquitination and stabilization of snail family transcriptional repressor 1, a transcription factor which is involved in the fibroblast activation (Díaz 2018). Activated fibroblasts initiate and enhance angiogenesis through the generation of vascular endothelial growth factor, TGF-β and platelet-derived growth factor (Newman et al. 2011). Therefore, USP27X may contribute to the pathogenesis of autoimmune fibrotic diseases such as scleroderma (Gilbane et al. 2013).

## DEAD-box helicase 3 X-linked (DDX3X)

DDX3X is a member of RNA helicases from DEAD-box family containing the conserved motif of Asp-Glu-Ala-Asp (DEAD motif). The family members are basically known for their functions in RNA metabolisms, including RNA transcription, processing, splicing and translation (Cordin et al. 2006). Recently, they have been identified as the immune sensors in innate immunity (Taschuk and Cherry 2020). Retinoic-acid inducible gene I, a cytosolic member of the pattern recognition receptors, is the most well-known member of the family implicated in the recognition of viral RNA in cooperation with two other proteins, including melanoma differentiation-associated 5 and laboratory of genetics and physiology 2 (An 2017).

Several recent studies addressed a role for DDX3X in the antiviral immune responses through induction of type I IFNs. Chicken DDX3X was detected to induce IFN-β through STING-TBK1-IRF7-IFN-β axis (a detail for this type of the signaling cascade has been presented in the DDX3X section. Moreover, DDX3X was able to increase generation of proinflammatory cytokine, including IL-1β, IL-6, and IL-8 as well as myxovirus resistance 1 and protein kinase R (Niu et al. 2019), two of the most studied antiviral IFN-stimulated genes which are implicated in the antiviral response of IFNs (Schoggins 2014). Porcine DDX3X was also induced IFN-β expression. More investigations showed DDX3X is co-localized with TBK1 and IKK suggesting involvement of these proteins in the signaling pathway of DDX3X (Chen 2014). Accordingly, fish DDX3X exerted an antiviral effect mediated through the activation of IRF3 and IRF7 transcription factors resulting in the production of IFN-β (Liu 2017). In addition to affecting IFN-β production, Samir et al. observed the lower activation of the NLR family pyrin domain containing 3 (NLRP3) inflammasome in the DDX3X deficient bone marrow-derived macrophages. This finding indicated the contribution of DDX3X in the NLRP3 inflammasome activation. Inflammasome activation stimulates inflammatory response through generation of the active IL-1β and interleukin-18 (Samir 2019).

Interestingly, a DDX3X dependent sex-biased activation of macrophages was detected in the macrophages exposed to *Listeria monocytogenes* infection. An approximate of 75% of the genes that transcriptionally altered upon the infection of the male wild-type macrophages, was responded to the infection in the DDX3X deficient counterparts. On the other hand, none of the altered genes in the female wild-type macrophages, were induced upon the infection in the female DDX3X deficient macrophages. A reduction in the expression level of the cytokines, including IL-1, IL-6, IL-12 and TNF-α as well as the chemokines was observed in the DDX3X deficient infected macrophages. In accordance with the previous studies, a reduction in the IFN-β level was also observed. The signaling pathways leading to the activation of IRF3/IRF7 and NF-κB transcription factors, were implicated in the cytokine production. Examination of DDX3X deficiency in the TLR signaling, exhibited an impaired response of the DDX3X deficient macrophages to the stimulation with TLRs agonist, including poly (I:C), TLR3 agonist, and lipopolysaccharide (LPS), TLR 4 agonist. Altogether, these findings suggest the implication of DDX3X in the TLR signaling and production of proinflammatory cytokines and chemokines (Szappanos 2018).

Despite the lack of information on the association between DDX3X and autoimmune diseases and according to the current findings, the DDX3X may contribute to the pathogenesis of autoimmune diseases and female bias of autoimmunity; however, further studies are necessary to address these issues.

## Chromosome X open reading frame 21 (CXORF21)

CXORF21 is an uncharacterized protein which has been recently identified as a candidate gene implicated in the female bias in SLE (Odhams 2019). A higher upregulation of CXORF21 protein in LPS and IFN-γ-stimulated monocytes and IFN-α-stimulated B cells was detected in the females than the males. In addition, the binding sites for NF-κB, STAT1, STAT2, STAT3 and IRF3 transcription factors were identified at the promoter of CXORF21 suggesting transcriptional regulation of CXORF21 by the cytokines (Odhams 2019). Accordingly, Harris et al. demonstrated CXORF21 implication in the female bias observed in SLE (Harris 2019). Interestingly, it was demonstrated that CXORF21 gene was transcriptionally inactive in the non-immune cells, whereas its transcripts were detected in the immune cells with the highest expression level in monocytes, neutrophils and IFN-α B cells. The magnitude of CXORF21 expression was higher in females than males (Odhams 2019). The authors in another study, reported the greater level of CXORF21 in the females immune cells than those of the males. An elevation in the CXORF21 protein was detected after TLR7 activation. In accordance, CXORF21 deficient monocytes were unable to increase the expression level of IFN-α following TLR7 stimulation. Therefore, CXORF21 protein may be involved in the female bias of SLE, acting downstream of TLR7 and regulate production of IFN-α. Accordingly, co-localization of the CXORF21 protein with TLR7 was also observed by the others (Paul 2013; Wu et al. 2015). Moreover, the lower endolysosomal pH was observed in the APCs from the females than that of the males. This suggests CXORF21 may regulate the antigen presentation (Harris et al. 2019), as the lower endolysosomal pH is in favor of the antigen processing in the endolysosomal compartment and thus subsequent antigen presentation on the cell surface (Abbas et al. 2017).

## X-linked inhibitor of apoptosis protein (XIAP)

Apoptosis is a physiologic cell death in which DNA, RNA and organelles are degraded and packed into the small vesicles called apoptotic bodies. Apoptotic bodies are quickly clear from the body by macrophages. Apoptosis is triggered through two pathways mediated by the enzymes called caspases: (1) Extrinsic pathway in which activation of the death receptors, including Fas receptor (CD95) and TNF receptor 2, triggers activation of caspase 8. (2) Intrinsic pathways in which the released cytochrome c from the membrane-ruptured mitochondria triggers activation of caspase 9. Activation of the initiator caspases, including caspase 8 and 9, are followed by the activation of the effector caspases (caspase 3, 6 and 7). The effector caspases activate a complex of the enzymes resulting in the degradation of DNA, cytoskeletal and nuclear proteins and formation of apoptotic bodies (Elmore 2007).

Activation of the caspases is tightly regulated through the inhibitors of apoptosis (IAP) proteins. XIAP is the best characterized protein of IAPs family and contains three Baculovirus IAP repeat (BIR) domains and a new gene finger domain. The BIR domains are responsible for the interaction of XIAP with several caspases and inhibition of their activation. The BIR2 domain is known to be involved in the interaction with caspase 3 and 7, whereas the BIR3 domain is implicated in the binding to caspase-9. The high level of XIAP was associated with the poor responses of the tumor cells to the chemotherapy and other treatments (Flanagan 2015; Schiffmann 2019). Due to its roles in the prevention of apoptosis, XIAP has been a target of the immunotherapy in various cancer cells (Abbas and Larisch 2020; Katragadda et al. 2013; Holcik et al. 2001).

Autoimmunity may arise from the survival of autoreactive lymphocytes which are intended for the removal by the apoptotic death during central or peripheral tolerances. Autoreactive lymphocytes which are generated during the developmental stages, differentiation or activation processes, are deleted or inactivated by various tolerance mechanisms either in the central lymphoid organs (central tolerance) or in the peripheral organs (peripheral tolerance) (Abbas et al. 2017). Apoptosis plays a fundamental role in the deletion of autoreactive lymphocytes, therefore, any defects in this process contribute to the development of autoimmune diseases. Moreover, the activated lymphocytes massively expanded in the response to the antigens (or autoantigens), intended to be died by the apoptosis to keep the immune responses in the homeostatic state (Abbas et al. 2017).

XIAP deficient patients showed more apoptosis rate in their lymphocytes after receiving stimulator signals, including the activators of TCRs (anti-CD3), Fas receptors (anti-CD95), and TNF-related apoptosis-inducing ligand (TRAIL) receptors (tetrameric TRAIL molecules) (Rigaud 2006). In EAE, the animals overexpressed XIAP showed a severer form of the disease in comparison with the wild type animals which was associated with the resistance of T cells to the apoptosis (Moore 2008).

Despite using various stimulators of apoptosis for the induction of apoptotic cell death in the synovial tissues obtained from the patients with active RA, an inhibition in the cell death was observed. The phenomenon was associated with the elevated expression level of XIAP (Dharmapatni 2009). Synovial fluid´s lymphocytes of the patients with juvenile idiopathic arthritis also demonstrated a resistance to the apoptosis which was associated with the elevated level of XIAP (Smolewska et al. 2006). Therefore, XIAP can assist in the pathogenesis of autoimmune diseases. Female SLE T cells showed increased expression of XIAP compared to male SLE patients and female healthy controls suggesting a female-biased role of XIAP in SLE disease (Syrett et al. 2019). Interestingly, sex dimorphism in cerebral ischemia with a tendency towards female gender was attributed to XIAP (Dai and Ahmed 2014).

## Conclusion

Though XCI is applied to equalize the impact of two X chromosomes in females, however, some genes escape from XCI. The current literature provides evidence in the contribution of a number of the escape genes, including CD40L, CD99, LAMP-2, IRAK-1, TLR7, USP27X, DDX3X, CXORF21 and XIAP, in the autoimmunity. Reviewing present literature for the contribution of some escapes genes including CD40L, IRAK-1, TLR7, CXORF21 and XIAP confirms their primary attributions to the female bias of autoimmune diseases especially SLE disease. However, more studies are required to evaluate contribution of these genes in the female bias of SLE using animal model of the disease and the efficacy of therapeutic interventions against these molecules for example using monoclonal antibodies. In addition, next experiments are necessary to address attribution of these genes to the female bias of other autoimmune diseases rather than SLE. Finally, investigating role of the other escape genes such as USP27X and DDX3X in the sex-bias of autoimmune diseases such as SLE, RA, MS may help to discover further mechanisms involved in the female bias of autoimmunity and consequently in developing more effective treatments and sensitive diagnostics of patients with autoimmune diseases.

## Data Availability

Not applicable.

## Abbreviations

ABCs: Age-associated B cells
ABIN1: A20-binding inhibitor of NF-κB1
AIDCA: Activation-induced cytidine deaminase
AMP: Adenosine monophosphate
Anti-dsDNA: Anti-double stranded DNA antibodies
AP-1: Activator protein 1
APCs: Antigen presenting cells
Arl8b: ADP ribosylation factor like GTPase 8b
BCR: B cell receptors
BIR: Baculovirus IAP repeat
CCL5: Chemokine (C-C motif) ligand 5
CCND3: Cyclic D3
CD1a: Cluster of differentiation 1a
CD3: Cluster of differentiation 3
CD40: Cluster of differentiation 40
CD40L: Cluster of differentiation 40 ligand
CD80: Cluster of differentiation 80
CD86: Cluster of differentiation 86
CD99: Cluster of differentiation 99
cGAMP: Cyclic GMP-AMP
cGAS: GMP-AMP synthase
CMA: Chaperon mediated autophagy
CNS: Central nervous system
CTLs: Cytotoxic T lymphocytes
CXCL12: C-X-C motif chemokine 12
CXORF21: Chromosome X open reading frame 21
DCs: Dendritic cells
DDX3X: DEAD-box helicase 3 X-linked
DNA: Deoxyribonucleic acid
EAE: Experimental autoimmune encephalomyelitis
ERK: Extracellular signal-regulated kinase
FcγR: Fc gamma receptors
FDCs: Follicular dendritic cells
GCs: Germinal centers
GM-CSF: Granulocyte-macrophage colony-stimulating factor
GMP: Guanosine monophosphate
H2AK119ub: H2AK119 mono-ubiquitination
H3K27me3: H3K27 trimethylation
IAP: Inhibitors of apoptosis
IBDs: Inflammatory bowel diseases
IFN-α: Interferon-alpha
IFN-β: Interferon-beta
IFN-γ: Interferon-gamma
IKK: IκB kinase
IL-1: Interleukin-1
IL-10: Interleukin-10
IL-12: Interleukin-12
IL-1β: Interleukin-1 beta
IL-2: Interleukin-2
IRAK-1: Interleukin-1 receptor-associated kinase-1
IRF3: Interferon regulatory factor 3
IRF7: Interferon regulatory factor 7
IκB: Inhibitor of NF-κB activation
JNK: C-Jun N-terminal kinase
LAMP-2: Lysosome-Associated membrane Protein 2
LPS: Lipopolysaccharide
MAPK: Mitogen-activated protein kinase
MHC I: MHC class I
MHC II: MHC class II
MHC: Major histocompatibility complex
MMP13: Matrix metalloproteinase 13
MMP9: Matrix metalloproteinase 9
mRNA: Messenger ribonucleic acid
MS: Multiple sclerosis
MyD88: Myeloid differentiation primary response 88
NFAT: Nuclear factor of activated T cells
NF-κB: Nuclear factor kappa-light-chain-enhancer of activated B cells
NLRP3: NLR family pyrin domain containing 3
PBMCs: Peripheral blood mononuclear cells
pDCs: Plasmacytoid dendritic cells
Plekhm2: Pleckstrin homology domain-containing family M member 2
PRC: Polycomb repressive complexes
RA: Rheumatoid arthritis
SLE: Systemic lupus erythematosus
ssRNA: Single-stranded RNA
STAT3: Signal transducer and activator of transcription 3
STING: Stimulator of interferon genes
TAK1: TGF-β-activated kinase 1
TBK1: TANK binding protein 1
TGF-β: Transforming growth factor-beta
Th: T helper
Th1: T helper 1
Th2: T helper 2
TLR7: Toll-like receptor 7
TLRs: Toll-like receptors
TNF: Tumor necrosis factor
TNF-α: Tumor necrosis factor-alpha
TRAF: TNF receptor-associated factor
TRAIL: TNF-related apoptosis-inducing ligand
Tregs: Regulatory T cells
USP27X: Ubiquitin carboxyl-terminal hydrolase 27
XCI: X chromosome inactivation
XIAP: X-linked inhibitor of apoptosis protein
XIC: X-inactivation center
XIST: X-inactive specific transcript
